# Tick-borne encephalitis (TBE) cases are not random: explaining trend, low- and high-frequency oscillations based on the Austrian TBE time series

**DOI:** 10.1186/s12879-020-05156-7

**Published:** 2020-06-26

**Authors:** Franz Rubel, Melanie Walter, Janna R. Vogelgesang, Katharina Brugger

**Affiliations:** Unit for Veterinary Public Health and Epidemiology, University of Veterinary Medicine Vienna, Austria, Veterinaerplatz 1, Vienna, 1210 Austria

**Keywords:** Vector-borne disease, Climate change, Scandinavian index, Beech fructification, Mast seeding, Prediction, Ixodes ricinus

## Abstract

**Background:**

Why human tick-borne encephalitis (TBE) cases differ from year to year, in some years more 100%, has not been clarified, yet. The cause of the increasing or decreasing trends is also controversial. Austria is the only country in Europe where a 40-year TBE time series and an official vaccine coverage time series are available to investigate these open questions.

**Methods:**

A series of generalized linear models (GLMs) has been developed to identify demographic and environmental factors associated with the trend and the oscillations of the TBE time series. Both the observed and the predicted TBE time series were subjected to spectral analysis. The resulting power spectra indicate which predictors are responsible for the trend, the high-frequency and the low-frequency oscillations, and with which explained variance they contribute to the TBE oscillations.

**Results:**

The increasing trend can be associated with the demography of the increasing human population. The responsible GLM explains 12% of the variance of the TBE time series. The low-frequency oscillations (10 years) are associated with the decadal changes of the large-scale climate in Central Europe. These are well described by the so-called Scandinavian index. This 10-year oscillation cycle is reinforced by the socio-economic predictor net migration. Considering the net migration and the Scandinavian index increases the explained variance of the GLM to 44%. The high-frequency oscillations (2–3 years) are associated with fluctuations of the natural TBE transmission cycle between small mammals and ticks, which are driven by beech fructification. Considering also fructification 2 years prior explains 64% of the variance of the TBE time series. Additionally, annual sunshine duration as predictor for the human outdoor activity increases the explained variance to 70%.

**Conclusions:**

The GLMs presented here provide the basis for annual TBE forecasts, which were mainly determined by beech fructification. A total of 3 of the 5 years with full fructification, resulting in high TBE case numbers 2 years later, occurred after 2010. The effects of climate change are therefore not visible through a direct correlation of the TBE cases with rising temperatures, but indirectly via the increased frequency of mast seeding.

## Background

The tick-borne encephalitis (TBE) virus is a flavivirus persisting in a natural transmission cycle between small mammals and ticks. Humans can be infected, but they are ecologically dead-end hosts [1]. TBE vectors in Central Europe are predominantly ticks of the genus *Ixodes*, especially *Ixodes ricinus*, the castor bean tick [2]. Since TBE can be a serious disease in humans [3], it is notifiable in almost all endemic TBE areas. Despite the availability of efficient vaccines [4, 5], TBE cases in Central Europe has risen sharply in recent decades [6]. In 2018, historical maximum values of 584 cases in Germany [7] and 377 cases in Switzerland [8] were registered. In Austria, 154 cases were the highest reported since 1994, although more than 80% of the population is vaccinated [9, 10]. Without vaccination probably more than 800 TBE cases per year would occur in Austria. Looking at the long time series of TBE cases, some of which date back to the 1950s [11], the question arises of how the temporal variations of these TBE cases can be explained. It can be taken into account that climate and environmental variables, averaged over large areas such as Central Europe, explain biological relationships much better than those with high local accuracy, as discussed in the fundamental papers on patterns and scales in ecology from Levin [12] and Hallett et al. [13]. Additionally, it can be taken into account that different mechanisms act on different time scales.

For example, long-term TBE trends, that have been observed over many decades, have been linked to factors such as demographic trends, changes in land use and associated wildlife density, or changes in human recreational behavior and related exposure [14]. Not least, climate change has been discussed as a possible driver [15, 16]. While in Sweden TBE incidence was significantly related to milder winters and higher spring and autumn temperatures [17], for the Baltic countries it was stated that climate change cannot explain the increase in TBE cases [18]. Here, it is assumed that climate change plays a only minor role in explaining the trend of Austrian TBE cases. Instead, the demographic development of the population is assumed to be the most probable cause for the rising TBE trend. This long-term trend in the Austrian TBE time series is superimposed by cyclical fluctuations. The duration period of these cyclical fluctuations was determined by Zeman [19] for 6 time series of TBE cases in Austria, the Czech Republic, the German federal states Bavaria and Baden-Wuerttemberg, Slovenia, and Switzerland. Calculating the power spectra from the detrended time series of Austrian TBE cases results in 2 dominant periods of the oscillations. The first has a period of 10 years (low-frequency oscillations), the second has a period of 2–3 years (high-frequency oscillations) [19].

It is well-known that the large atmospheric circulation variability is responsible for population and disease fluctuations [20]. Atmospheric circulation variability is also referred to as climate variability and is often described by so-called teleconnection indices. The best known of these climate variability or anomaly indices is the El Niño Southern Oscillation (ENSO), which occurs in areas around the tropical Pacific, especially in the southern hemisphere. ENSO triggered Malaria, Dengue, Rift Valley fever and other vector-borne disease outbreaks [21]. The ENSO impact on outbreaks reaches as far as the south of the USA, where a rodent-borne hantavirus outbreak was associated with the 1997–1998 El Niño [21]. The most studied climate variability of the northern hemisphere is the North Atlantic Oscillation (NAO). It has been linked to a variety of disease outbreaks in the USA and Western Europe [22].

For example, Hubálek [23] studied 14 viral, bacterial and protozoan notifiable human diseases in the Czech Republic and their association with NAO indices, but no correlation was found for the tick-borne diseases TBE and Lyme borreliosis. Palo [24] also found no correlation between NAO and the number of Swedish TBE cases. Another teleconnection index describing the large-scale atmospheric circulation variability is the Scandinavian index (SCAND). It is less known than ENSO and NAO, and there is currently only one study that correlates human disease data, the UK asthma mortality, with SCAND fluctuations [25]. Since SCAND describes the large-scale atmospheric circulation variability from Central Europe to Central Asia, it is hypothesized that it is suitable for describing the 10-year oscillations in TBE cases in Austria.

The 2–3 year oscillations might be caused by the variations in beech fructification, which is responsible for the population dynamics of small mammals [26]. This also describes the oscillations in the population of *I. ricinus* whose larvae and nymphs feed mainly on yellow-necked mice *Apodemus flavicollis* and bank voles *Myodes glareolus* [27] and thus contribute to the natural TBE virus transmission cycle. Brugger et al. [28] demonstrated that with the beech fructification index 2 years prior, the annual average temperature of the previous year and the past winter temperature, the *I. ricinus* nymphal density can be described with great accuracy. However, some peaks in TBE time series cannot be explained by tick density. An example are the extraordinary high TBE numbers in 2006, which were observed in some European countries (not in Austria). They were explained by recreational behavior of humans, i.e. more outdoor activities in the extremely warm year 2006 [29]. Because there are no long-term studies, a simple hypothesis is pursued, according to which more sunshine hours should lead to more outdoor activities and thus to a higher exposure of the human population.

Using the Austrian time series, we aimed at identifying the demographic and environmental factors associated with the trend and the oscillations by using generalized linear models (GLMs). Both the observed and the predicted TBE time series are to be subjected to a spectral analysis. The resulting power spectra should then indicate which predictors are responsible for the trend, the high-frequency and the low-frequency oscillations, and with which explained variance they contribute to the TBE oscillations. The model development presented here differs fundamentally from the usual approaches, according to which significant predictors are selected from a large number of possible predictors by stepwise modeling [30], whereby their contribution to the frequency spectrum is not taken into account. So far, only two GLMs have been developed to predict TBE time series. The first is a GLM to predict the numbers of Swedish TBE cases by using December precipitation and red fox (*Vulpes vulpes*) or mink (*Mustela vison*) abundance as predictors [31]. The second GLM confirmed the high correlation between red fox density and TBE cases [24], which underpin the causal link between beech fructification, small mammals, and their predators, red foxes and mink.

## Methods

**Fig. 1:**
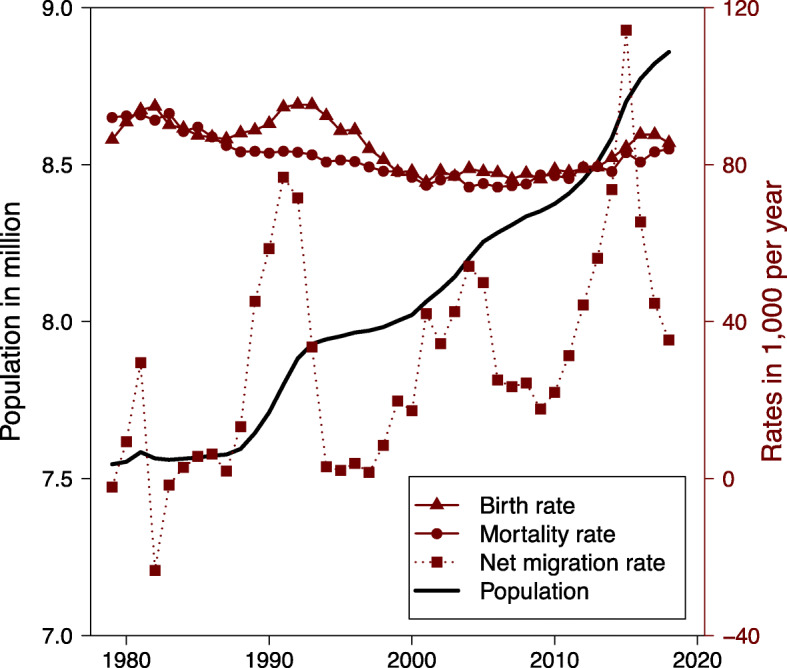
Demographics of the Austrian human population. Left axis: population in million (black line), right axis: birth rate, mortality rate and net migration rate in 1,000 per year (red lines). Noteworthy is the net migration, which is exclusively responsible for the population growth. Period 1979–2018

Two ways of representing annual TBE time series are in use. On the one hand the absolute number of annual TBE cases is indicated, on the other hand the TBE incidence, i.e. the number of annual TBE cases per 100,000 inhabitants. Here, absolute numbers of annual TBE cases are used to allow the demographic parameters of the human population (Fig. 1) to be used as predictors. Thus, for example, the variance of the TBE time series explained by the population growth can be determined.

### Demographics of the human population

Since the Austrian population has risen sharply in recent years, the demographic development must be taken into account. It is described by the birth rate, the mortality rate, and the net migration rate. Figure 1 shows the official demographic data [32]. According to this, the human population increased by more than 1.2 million in the period 1979–2018. This is mainly due to the net migration rate, i.e. the difference between annual immigration and emigration. Four major net migration (immigration) events occurred within the 40-year period 1979–2018. The 2 most outstanding immigration events were caused by the Yugoslavian Civil War in 1991 and the Syrian Civil War in 2015. Net migration peaks were also observed 1981 after the suppression of the anti-communist social movement Solidarnosc in Poland and during 2001–2005 after the labor market has been opened further [33]. The difference between the birth rate and the mortality rate, the reproduction rate, is on average just 3,000 people per year. This is one order of magnitude lower than the mean net migration rate of about 30,000 people per year. Here, the total population *N*_*TOT*_ and the net migration *N*_*MIG*_ were used as predictors and listed in Table 1.

**Table 1:** Input data and model output for the period 1979–2018: total human population *N*_*TOT*_ in 10^6^, annual human net migration *N*_*MIG*_ in 10^4^ per year, reported tick-borne encephalitis (TBE) cases *N*_*TBE*_, vaccination coverage *VC*, four-year average of log-transformed Scandinavian indices *SI*, beech fructification index 2 years prior *F*_*year*−2_ and annual sunshine duration in hours *SD*

Year	*N*_*TOT*_	N _*MIG*_	*N*_*TBE*_	*VC*	*SI*	*F*_*year*−2_	*SD*	*N*	*N*_*GLM*1_	*N*_*GLM*2_	*N*_*GLM*3_	*N*_*GLM*4_
1979	7.546	-0.002	677	0.03	0.974	1	1447	698	436	652	645	634
1980	7.553	0.009	438	0.07	0.904	1	1444	471	437	526	531	512
1981	7.584	0.030	294	0.15	0.836	1	1392	346	440	411	419	385
1982	7.564	-0.023	612	0.22	0.801	1	1719	785	438	508	546	602
1983	7.560	-0.002	240	0.29	0.725	0	1583	338	437	380	305	318
1984	7.563	0.003	336	0.35	0.794	2	1401	517	438	432	473	439
1985	7.567	0.006	300	0.41	0.824	1	1522	508	438	454	474	469
1986	7.573	0.006	258	0.46	0.804	2	1555	478	439	435	473	478
1987	7.576	0.002	215	0.51	0.837	0	1371	439	439	480	368	349
1988	7.594	0.013	201	0.56	0.744	2	1451	457	441	370	411	386
1989	7.645	0.045	131	0.60	0.722	2	1732	328	447	302	331	357
1990	7.711	0.059	89	0.63	0.714	0	1692	241	455	283	220	237
1991	7.799	0.077	128	0.65	0.728	2	1679	366	465	274	296	305
1992	7.883	0.071	84	0.67	0.759	2	1623	255	475	314	338	338
1993	7.929	0.034	102	0.71	0.815	1	1539	352	481	449	471	453
1994	7.943	0.003	178	0.74	0.859	3	1612	685	483	591	660	640
1995	7.953	0.002	109	0.78	0.848	0	1601	495	484	582	457	477
1996	7.965	0.004	128	0.78	0.899	1	1504	582	485	648	675	650
1997	7.971	0.002	99	0.79	0.767	3	1722	471	486	494	573	577
1998	7.982	0.008	62	0.80	0.704	0	1438	310	487	416	343	318
1999	8.002	0.020	41	0.82	0.732	0	1626	228	490	419	340	348
2000	8.021	0.017	60	0.84	0.695	1	1546	375	492	395	442	414
2001	8.064	0.042	54	0.86	0.783	1	1514	386	498	425	452	421
2002	8.100	0.034	60	0.87	0.871	2	1533	462	502	546	588	566
2003	8.143	0.043	82	0.87	0.873	1	2014	631	508	534	552	681
2004	8.201	0.054	54	0.87	0.840	1	1589	415	516	478	498	482
2005	8.254	0.050	100	0.88	0.862	1	1743	833	523	526	547	576
2006	8.283	0.025	84	0.88	0.872	2	1771	700	526	627	688	743
2007	8.308	0.023	45	0.88	0.827	0	1692	375	530	580	462	487
2008	8.335	0.024	87	0.87	0.837	2	1635	669	533	597	666	661
2009	8.352	0.018	79	0.86	0.835	1	1686	564	536	622	674	682
2010	8.375	0.022	63	0.85	0.837	0	1538	420	539	616	492	476
2011	8.408	0.031	113	0.86	0.889	3	1847	807	544	664	741	785
2012	8.452	0.044	52	0.85	0.885	0	1674	347	550	625	484	503
2013	8.508	0.056	99	0.82	0.880	3	1508	550	558	593	656	569
2014	8.585	0.074	80	0.85	0.892	0	1621	533	569	571	436	433
2015	8.700	0.114	71	0.85	0.828	1	1743	473	586	417	431	428
2016	8.773	0.065	89	0.84	0.807	1	1607	556	597	542	589	543
2017	8.822	0.045	116	0.82	0.772	0	1596	644	604	577	478	451
2018	8.859	0.035	154	0.82	0.747	3	2015	856	610	586	708	768

The hypothetical TBE cases without vaccination *N* were simulated by 4 versions (development steps) of a generalized linear model, where the predicted TBE cases are given by N _*GLM*1_, N _*GLM*2_, N _*GLM*3_ and N _*GLM*4_

### Human TBE time series

The official human TBE time series in Austria for the period 1979–2018 is analyzed. This 40-year time series was documented by the Department of Virology, Medical University of Vienna, acting as the national reference laboratory for TBE virus infections. Only hospitalized patients with a serologically confirmed recent infection with TBE virus were counted as cases and published together with the vaccination coverage of the Austrian population [34]. In Austria, TBE is a notifiable disease and thus accuracy of the records is very high. Using the official vaccination coverage a hypothetical time series without TBE vaccination was estimated.

$$ N = \frac{1}{1 -{VC}} \; N_{{TBE}} $$

Here, *N*_*TBE*_ are the annual TBE cases documented by the national reference laboratory for TBE virus infections, *VC* is the official vaccination coverage within the interval [0, 1], and *N* is the hypothetical TBE cases without vaccination. Values of *N*_*TBE*_, *VC* and *N* are listed in Table 1. In the following, only the hypothetical TBE cases *N* are used to investigate the natural trend and the oscillations in the Austrian TBE time series.

### Climate teleconnection

To describe the decadal changes of the large-scale climate in Central Europe several teleconnection indices are available. Here, the Scandinavian index (SCAND) developed by Barnstone and Livezey [35] was used, which the authors originally called the Eurasia-1 pattern. With the help of SCAND an atmospheric circulation pattern, i.e. the spatial arrangement of northern hemisphere high- and low-pressure systems, is characterized by a single index value. Like ENSO and NAO, the SCAND is therefore well suited for investigating correlations of large-scale atmospheric circulation patterns with the cases of vector-borne diseases. A time series of the monthly SCAND is provided for the period 1950 to present on the Climate Prediction Center (CPC) website of the National Oceanic and Atmospheric Administration [36].

**Fig. 2:**
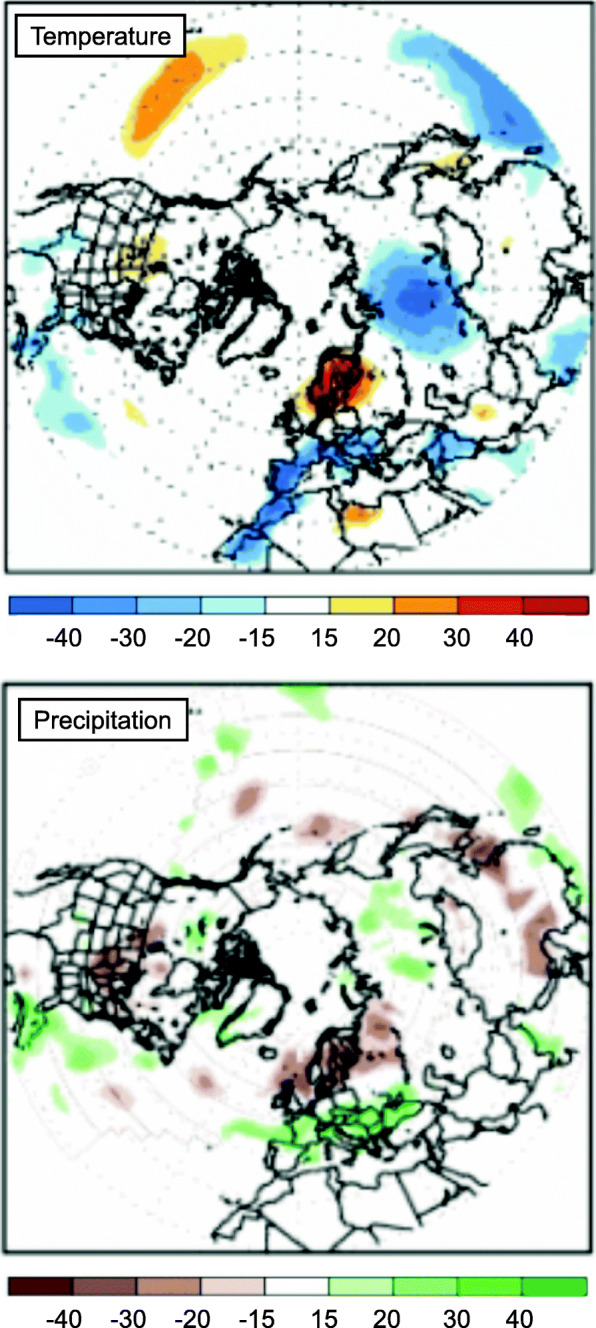
Maps showing correlation between the high July values of the Scandinavian index (SCAND) and the monthly climate anomalies during June, July, and August. Maps of temperature and precipitation anomalies (departures from mean in percent) were adapted from NOAA [36]

The SCAND describes an atmospheric circulation center over Scandinavia, with weaker centers of opposite sign over western Europe and eastern Russia/western Mongolia. Positive values are associated with below-average temperatures across western Europe and central Russia. It is also associated with above-average precipitation across central and southern Europe and below-average precipitation across Scandinavia (Fig. 2). For the TBE endemic areas in Austria and southern Germany, this means that high SCAND values represent cooler and rainier periods. In turn, low SCAND values describe above-average warm and dry periods.

To determine the optimal correlation between TBE and SCAND, so-called cross-correlation maps (CCMs) were used. With CCMs optimal time lags and accumulation periods of predictors can be determined [37]. As known from vector biology, the best correlation between arthropod vectors or disease cases caused by pathogens they transmit and environmental temperature is obtained when temperature data were averaged over the period of the life cycle of the vector. For example, the life cycle of *Culex pipiens*, the vector of West Nile virus, is about 2–3 weeks during the mosquito activity period. With CCMs, 18 days were determined as the optimal averaging period [38]. If one wants to describe the dynamics of the Bluetonge virus vector *Culicoides obsoletus* by temperature, the somewhat longer averaging period of 37 days was estimated according the typical length of the life cycle of biting midges [39]. For TBE and its main vector *I. ricnus* the averaging period of the climate predictor SCAND should therefore be 2–6 years [40], 3–6 years [41], or 4–6 years [42]. In fact, the optimal averaging period determined by the application of CCMs is 4 years. Ideally, predictors should be normally distributed, which frequently can be achieved with a log-transformation. Therefore, the Scandinavian index *SI* used here is derived from the log-transformed monthly values of SCAND [36], which were averaged over 4 years. The *SI* values are listed in Table 1.

### Beech fructification

The natural transmission cycle of the TBE virus depends on the availability of suitable hosts for the main vector *I. ricinus*. Preferred hosts of *I. ricinus* larvae are among others small rodents [43]. As no observations of small rodents are available for the long time series investigated in this study, the fructification index of the European beech (*Fagus sylvatica*) was applied for indicating the rodent density. Beechnuts are a basic food source for small rodents resulting in population peaks one year after mast seeding [44, 45]. Higher host densities cause higher densities of larvae of the TBE vectors *I. ricinus*. Two year after mast seeding, higher densities of *I. ricinus* nymphs are observed [28], which may be responsible for peaks in human TBE time series. Since the mast seeding is continental-scale synchronized [46], only a single time series, the beech fructification index published by Konnert et al. [47], is used here. This index is defined as the annual seed production and is divided in the following four classes: (0) absent, i.e. no fructification, (1) scarce, i.e. sporadic occurrence of fructification, but not noticeable at first sight, (2) common, i.e. clearly visible fructification, and (3) abundant, i.e. full fructification, also known as mast seeding. The values of the beech fructification indices 2 years prior *F*_*year*−2_ are listed in Table 1.

### Annual sunshine duration

It is hypothesized that human recreational behavior affects the number of TBE cases. For example, higher outdoor activities increase exposure and they should therefore increase the TBE cases. Since there is no established predictor for human outdoor activities, the annual sunshine duration in hours is used as such here. With large-scale considerations in focus, this should be representative for Central Europe. Of all meteorological services in Central Europe, only the German Weather Service offers such open data [48]. This is averaged over the entire region of Germany and should also be representative of the smaller neighboring countries such as Austria. Thus, in addition to the averaged log-transformed Scandinavian index *SI*, and the beech fructification index 2 years prior *F*_*year*−2_, the annual sunshine duration *SD* is the third large-scale predictor used for the analysis of TBE time series (Table 1).

### Statistical modelling

All statistical analysis and modeling was done with the Language and Environment for Statistical Computing R [49]. GLMs were used to describe relationships. In the course of this, an overdispersion was observed since the dispersion parameter was generally greater than 1. This overdispersion was taken into account by using negative binomial models implemented with the R package mass [50].

To assess the necessary conditions for the application of GLMs, the predictors used (total population, net migration, Scandinavian index, beech fructification index, and annual sunshine duration) were tested for collinearity. This test is commonly used to select from a large number of predictor variables those that are most strongly correlated with the target variable, here the TBE cases. In addition, the so chosen predictor variables should be only weakly correlated with each other. Otherwise, their number can be further reduced. Here, however, a different approach is pursued: a small number of biologically well interpretable predictors are given. The check for collinearities (Fig. S1) therefore has only a control function, all correlations between the individual predictors are well below the threshold of |*R*|=0.7 [51]. With the R package psych [52] additional model diagnoses were created. These include examining the model errors for randomness (residual vs. fitted plot, scale location plot) and normal distribution (normal Q–Q plot), both of which are prerequisites for the applicability of GLMs [53]. Cook’s distance (residual vs. leverage plot) was used to test which TBE observations have the greatest influence on the regression. Outliers can be defined and eliminated if necessary [53], but this was not applied here.

While the statistical methods described above are intended to ensure the reliability of the selected model, it is particularly interesting how well the annual TBE fluctuations are described by the chosen predictor variables. Therefore, the models were verified by the root-mean-square error (RMSE) and the explained variance (R^2^). The advantage of these verification measures is that with the RMSE the error is specified in units of the target variable, i.e. the TBE cases, and R^2^ is well known.

If different GLMs are developed, the best model can be chosen with the help of the Akaike information criterion (AIC). The AIC estimates the quality of each model, relative to each of the other models. For better interpretability, however, the adjusted R^2^ (R$^{2}_{{adj}}$) is given here. In general, the use of additional predictors in GLMs leads to higher R^2^ values, even if they do not make a significant contribution to the model. With R$^{2}_{{adj}}$ this is considered, which also determines model performance, relative to each of the other models [53].

A key objective of this study is to describe the causes of the trend as well as the low-frequency and the high-frequency oscillations of the TBE cases. For this purpose, the power spectra of both the observed and the predicted TBE cases are calculated as described e.g. by [54]. The predictors for the GLMs should be selected so that the power spectra of the predictions match those of the observations as closely as possible.

## Results

In 4 steps GLMs (negative binomial regression models) were developed, which demonstrate the influence of the selected predictors on the model performance as well as on the power spectrum of the predicted TBE time series. Thus, a final model was stepwise developed, which explains more than two-thirds of the variance in the observations.

**Fig. 3:**
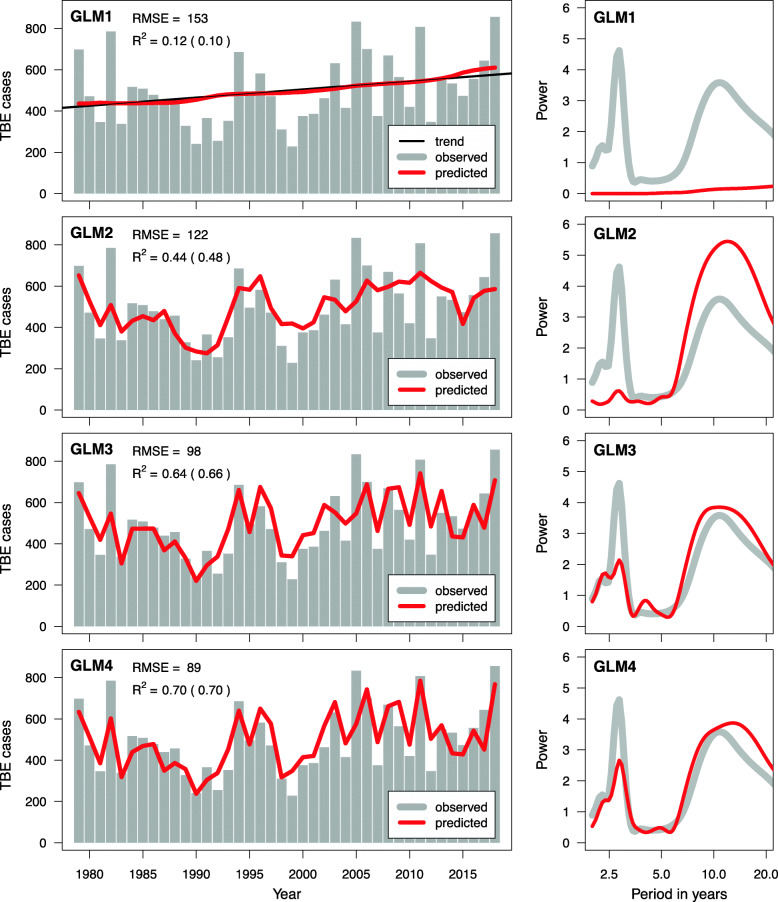
Observed (grey bars) and predicted (red lines) Austrian tick-borne encephalitis series (left) and corresponding power spectra (right). GLM1: model using exclusively the human population *N*_*TOT*_ as predictor variable resulting in a good approximation of the linear trend depicted by the black line. GLM2: model extended by the predictors net migration rate *N*_*MIG*_ and Scandinavian index *SI* to explain low-frequency oscillations. GLM3: model extended by the beech fructification index 2 years prior *F*_*year*−2_ to explain also high-frequency oscillations. GLM4: best performance model extended by the annual sunshine duration *SD*. For each model the verification measures root-mean-square error (RMSE) and explained variance R^2^ (with R$^{2}_{{adj}}$ in brackets) are given. Period 1979–2018

The first GLM uses only one predictive variable, the total population *N*_*TOT*_. Figure 3(GLM1) shows the TBE cases without vaccination *N* (grey bars) with the predicted TBE cases *N*_*GLM*1_ (red line) representing a good approximation of the linear trend calculated from the observations *N* (black line). A rank-order correlation coefficient after Spearman of R=0.29 between the total population and the observed TBE cases was estimated (Fig. S1). The corresponding power spectrum for the observations shows 2 maxima. The first one is located at a period of 3 years (high-frequency oscillations), the second one is located at a period of 10 years (low-frequency oscillations). The power spectrum of the model shows no maximum but only red noise, as expected for the trend.

The second GLM was extended to explain the low-frequency oscillations in addition to the trend. Additional predictors used were the transformed Scandinavian index *SI* and the annual net migration *N*_*MIG*_. Both contribute to the 10-year TBE oscillation. The rank-order correlation between the TBE cases and the *SI* was R=0.52 (Fig. S1). In Austria, periods of high *SI* are related to relatively cool summers with above-average precipitation (Fig. 2). Another highly significant contribution to explain the TBE cases is provided by the Austrian net migration rate, which can be considered as an socio-economic predictor. The net migration *N*_*MIG*_ is negatively correlated with the numbers of the TBE cases (R=-0.14). This suggests that new arrivals are less exposed to TBE virus infections, although they are responsible for the long-term population growth and thus also for the long-term increase in TBE cases. Since there is no study on this topic so far, it is hypothesized that the majority of immigrants from abroad initially settle in big cities where they are less exposed to TBE foci. The model therefore reduces the overestimated TBE cases during random net migration events. Figure 3(GLM2) shows the extended GLM verified with an error of RMSE=122 TBE cases and an explained variance of R^2^=0.44 (R$^{2}_{{adj}}$=0.48). The power spectrum clearly shows that the 10-year oscillation of the observed TBE time series is well associated with the 2 predictors *SI* and *N*_*MIG*_.

The third GLM considers the beech fructification index 2 years prior *F*_*year*−2_ as an additional predictor for the high-frequency oscillations. The fact that the mast seeding 2 years prior has a high influence on the density of the main TBE vectors *I. ricinus* has already been shown by Brugger et al. [28]. Here it is demonstrated that this also applies to the TBE cases (R=0.38). Figure 3(GLM3) shows the model extended by the predictor beech fructification index *F*_*year*−2_. The power spectrum clearly shows that the fructification index contributes to the explanation of the high-frequency oscillations, although the power is slightly too low compared to the spectrum of the observed TBE cases. The period of the low-frequency oscillations, on the other hand, fits those of the observed TBE time series very well. The explained variance increases to R^2^=0.64 (R$^{2}_{{adj}}$=0.66), resulting in a further reduction of the error of RMSE=98 cases. Considering the uncertainties in the observed TBE cases and the fact that time series of disease cases are generally difficult to explain, this result can be classified as very good. The parameter estimates and the significance levels of the predictors of this GLM are summarized in Table 2, where the discrete values of the fructification index are modeled as a factor. The factor *F*_*year*−2_=0 is set as default and the factors *F*_*year*−2_=1, 2 or 3 are considered by different parameter estimates. Thus, the GLM requires only the four predictors *N*_*TOT*_,*N*_*MIG*_, *SI* and *F*_*year*−2_, all of which contribute to the model very significantly (p <0.001).

**Table 2:** Summary of GLM3, a negative binomial model for tick-borne encephalitis cases without vaccination (hypothetical cases)

	Estimate	SE	z	p	
Intercept	0.453	0.873	0.519	0.604	
*N*_*TOT*_·10^−6^	0.522	0.109	4.785	<0.001	***
*N*_*MIG*_·10^−6^	-6.389	1.467	-4.356	<0.001	***
*SI*	1.805	0.509	3.547	<0.001	***
*F*_*year*−2_=1	0.304	0.081	3.731	<0.001	***
*F*_*year*−2_=2	0.340	0.093	3.663	<0.001	***
*F*_*year*−2_=3	0.359	0.106	3.384	<0.001	***

The total population number *N*_*TOT*_, the net migration *N*_*MIG*_, the averaged log-transformed Scandinavian index *SI* as well as beech fructification factors 2 years prior *F*_*year*−2_=1, 2 or 3 were used as predictors for which the estimate, the standard error SE, the z-value (test statistics), and the *p*-value (significance) are given

The fourth GLM was extended by a predictor for the outdoor activity of humans. So far, no influence of human recreational behavior on the annual TBE time series has been considered, which should lead to a further improvement of the model. A climatic parameter that should have a plausible influence on an increased outdoor activity of humans is annual sunshine duration *SD* in hours. It is directly (without a lag time) correlated with the TBE cases (R=0.27). Thus, the correlation between *SD* and *N* is similar to the correlation between *F*_*year*−2_ und *N*. Since there is no appreciable collinearity between *SD* and *F*_*year*−2_ (Fig. S1), the consideration of *SD* results in an increased model performance. Figure 3(GLM4) shows the results of the GLM with the additional predictor *SD*. It explains the variations of the TBE cases even better, namely with R^2^=0.70 (R$^{2}_{{adj}}$=0.70), which leads to a further reduction of the error of RMSE=89 cases. The parameter estimates and the significance levels of the predictors of the final model are summarized in Table 3. Again, all predictors contribute significantly to model performance, with most *p*-values being very significant. Of course, the relative contribution of the fructification index decreases at the expense of sunshine duration, as both predictor variables are responsible for high-frequency oscillations. Statistical features for the final GLM as described in “Statistical modelling” section are provided in Fig. S2, the AIC values for the stepwise developed models in Table S1.

**Table 3:** Summary of GLM4, a negative binomial model for tick-borne encephalitis cases without vaccination (hypothetical cases)

	Estimate	SE	z	p	
Intercept	0.126	0.831	0.151	0.880	
*N*_*TOT*_·10^−6^	0.438	0.109	4.006	<0.001	***
*N*_*MIG*_·10^−6^	-6.589	1.381	-4.772	<0.001	***
*SI*	2.004	0.485	4.131	<0.001	***
*F*_*year*−2_=1	0.267	0.077	3.454	<0.001	***
*F*_*year*−2_=2	0.311	0.088	3.526	<0.001	***
*F*_*year*−2_=3	0.276	0.105	2.643	0.008	**
*SD* · 10^−3^	0.542	0.239	2.273	0.023	*

The total population number *N*_*TOT*_, the net migration *N*_*MIG*_, the averaged log-transformed Scandinavian index *SI* as well as beech fructification factors 2 years prior *F*_*year*−2_=1, 2 or 3 as well as the annual sunshine duration *SD* were used as predictors for which the estimate, the standard error SE, the z-value (test statistics), and the *p*-value (significance) are given

## Discussion

The climate of the TBE endemic areas in Austria and neighboring countries is still characterized by the boreal coniferous climate of the northern hemisphere in the 1960s. According to the well-known Köppen-Geiger climate classification it is known as Dfb climate, a boreal climate with rain at all seasons and warm summers [55]. Until today, this boreal coniferous climate has almost completely retreated from the Alps and has been replaced by a warm temperate Cfb climate [56]. It is called, according to the prevailing tree species in natural forests, beech climate. In order to take this into account, spruce monocultures threatened by climate change are being gradually replaced by deciduous or mixed forests across the region of the greater Alps. These more species-rich forests also provide better living conditions for the most important TBE virus vector *I. ricinus*, resulting in higher *I. ricinus* densities [57, 58]. Beech fructification is a predictor of the intensity of the natural TBE virus transmission cycle between small mammals and ticks, with a high beech fructification index increasing the population density of small mammals and of *I. ricinus* larvae one year thereafter. One more year later, significantly higher densities of questing *I. ricinus* nymphs are responsible for the more frequent transmission of the TBE virus to humans [28]. However, effects on TBE cases due to these changes in forestry were only visible in the last decade, where higher frequencies of years with full fructification of beech (mast seeding) are responsible for several peaks in the TBE time series. A total of 3 of the 5 years with full fructification occurred after 2010 (Table 1). The effects of climate change are therefore not visible through a direct correlation of the TBE cases with rising temperatures, but indirectly via the increased frequency of mast seeding (*F*_*year*−2_). In combination with the rapidly increasing human population (N _*TOT*_, N _*MIG*_) and a slight decline in vaccination coverage (*VC*), this explains the major effects of rising numbers of Austrian TBE cases observed after 1995 under real conditions with vaccination (see *N*_*TBE*_ in Table 1). Additionally, 10-year oscillations are associated with the large-scale distribution of atmospheric high and low pressure systems (*SI*) resulting in the third model version GLM3, which explains 64% of the variation of Austrian TBE cases. In Austria, periods of high *SI* are related to relatively cool summers with above-average precipitation (Fig. 2). This may influence the density of the TBE vector *I. ricinus*, which does not like hot summers and extreme drought. Remarkably, oscillations with periods of 10 and 3–4 years were also observed for the TBE vector *Ixodes persulcatus* in Russia. Years with high tick densities follow frequently those with the peak population density of small mammals [41]. However, no direct correlation between human TBE cases and the TBE vectors *I. ricinus* and *I. persulcatus* has yet been published. Similar ecological connections are also known from the oak forests of eastern North America [44].

With the additional predictor sunshine duration *SD* in GLM4 the explained variance continues to increase. The hypothesis was that the exposure of the population increases with an increasing number of hours of sunshine. This hypothesis seems confirmed, as the explained variance in GLM4 increased to 70%. But that must not hide the fact that further studies on the contribution of human behavior to the cases of TBE are needed. It should be noted that GLM4 cannot be used for TBE forecasts because neither social behavior itself (e.g. during the unexpected SARS-CoV-2 pandemic 2020) nor *SD* can be estimated for the next 1–2 years. There is also a fraction of unexplained variance of 30%, which needs further research. In particular, rare extreme events are difficult to detect by statistics, because low case numbers result in low significance. For example, Dautel et al. [59] have shown for Germany that extreme low temperatures in January and February 2012, in combination with the lack of a protective snow cover, led to decreasing numbers of *I. ricinus* nymphs as well as very low numbers of human TBE cases in the same year (also recognizable in the Austrian TBE time series). The inclusion of this and similar field studies could help to improve future predictions.

Another aspect that has not been considered so far concerns the gender and age distribution of TBE cases within the population. The age distribution of the TBE cases shows a maximum at 55 years [3], with generally more men being infected with the TBE virus [60]. It has not yet been investigated whether the increasing number of TBE cases are related to the aging society.

It should also be noted that alternative predictors for the explanation of TBE cases are mentioned in the literature. In Slovenia, for example, a correlation was found between TBE cases and roe deer density 3 years ago [61]. A high roe deer (*Capreolus capreolus*) density is interpreted as a high host density for the TBE vector *I. ricinus* and consequently responsible for high TBE cases. For Austria, the results of Knap and Avs̆ic̆-Z̆upanc [61] could be confirmed, but the explained variance of the GLM4 decreased with the use of roe deer density instead of *SI* from 70% to 58%. This is because the correlation between the roe deer density and the TBE cases is largely due to the concurrent trends. The estimation of unknown wildlife densities from hunting index generally leads to some uncertainties. In addition, Austrian hunting data [62] from the statistical database STATcube provided by Statistics Austria [63] are not available in near real-time, as the hunting year differs from the calendar year. It covers the period from April 1 to March 31 of the following year. Wildlife data are therefore generally less well-suited as predictors than climate data, especially with regard to a possible forecast of the next year’s TBE cases.

## Conclusions

The TBE models presented here confirm the work of Hallett et al. [13] that large-scale indices can predict ecological processes very well, probably better than local weather and climate parameters. As the beech fructification indices of the years 2017 and 2018 are responsible for the TBE cases in 2019 and 2020, GLM3 can also be applied to forecast the TBE cases of the next 2 years. This is possible because for the forecast mainly the high-frequency oscillations caused by *F*_*year*−2_ are interesting. The predictors relevant to the trend and the low-frequency oscillations, on the other hand, can be extrapolated by simple methods such as persistence or linear interpolation. GLM3 is also applicable to the neighboring countries such as Germany or Switzerland using the same predictors, since the Scandinavian index is representative for all of Central Europe and also the beech fructification is large-scale synchronized. This has be examined in a follow-up study that was carried out during the review process of the paper presented here [64]. The verification with independent TBE cases from 2019 has demonstrated the good performance of the forecasts.

Finally, it should be noted that the findings presented here can subsequently be used to create process models of the type susceptible-infected-recovered (SIR). These models represent the highest stage of development in epidemiological modelling as, unlike statistical models, they map the dynamics of population health based on the underlying processes of disease transmission. A first SIR model on the dynamics of the Austrian human TBE cases was presented by Rubel [65].

## Supplementary information

**Additional file 1Additional file S1**. Frequency distributions (red bars) of the Austrian TBE incidence without vaccination *N* and the predictors used in the generalized linear models (GLMs): total human population *N*_*TOT*_, net migration *N*_*MIG*_, transformed scandinavian index *SI*, beech fructification index 2 years prior *F*_*year*−2_ and, annual sunshine duration in hours *SD*. the following rank-order correlations with *N* have been determined: r=0.29 (*N*_*TOT*_), r=-0.14 (*N*_*MIG*_), r=0.52 (*SI*), r=0.38 (*F*_*year*−2_), and r=0.27 (*SD*). maximum collinearity of r=0.59 (*N*_*TOT*_ vs. *N*_*MIG*_). **Additional file S2**. Statistical features of GLM4. **Additional file S3**. Akaike information criterion (AIC) and explained variance (R$^{2}_{{adj}}$) values for stepwise developed models GLM1–GLM4.

## Data Availability

The datasets used and/or analysed during the current study are available from the corresponding author on reasonable request.

## Abbreviations

TBE: Tick-borne encephalitis
GLM: Generalized linear model
ENSO: El Niño Southern Oscillation
NAO: North Atlantic Oscillation
SCAND: Scandinavian index
SI: Transformed Scandinavian index
VC: Vaccination coverage
SD: Sunshine duration
CCM: Cross- correlation map
RMSE: Root-mean-square error
AIC: Akaike information criterion
SIR model: Susceptible-infected-recovered model
