# Supplementary material for: Tick-borne encephalitis (TBE) cases are not random: explaining trend, low- and high-frequency oscillations based on the Austrian TBE time series
Source: BMC Infect Dis. 2020 Jun 26;20:448. doi: 10.1186/s12879-020-05156-7 (PMC7316636; doi:10.1186/s12879-020-05156-7)
Supplement: Supplementary file 1 — Additional file 1Additional file S1. Frequency distributions (red bars) of the Austrian TBE incidence without vaccination N and the predictors used in the generalized linear models (GLMs): total human population NTOT, net migration NMIG, transformed scandinavian index SI, beech fructification index 2 years prior Fyear−2 and, annual sunshine duration in hours SD. the following rank-order correlations with N have been determined: r=0.29 (NTOT), r=-0.14 (NMIG), r=0.52 (SI), r=0.38 (Fyear−2), and r=0.27 (SD). maximum collinearity of r=0.59 (NTOT vs. NMIG). Additional file S2. Statistical features of GLM4. Additional file S3. Akaike information criterion (AIC) and explained variance (R$^{2}_{{adj}}$adj2) values for stepwise developed models GLM1–GLM4. [file 12879_2020_5156_MOESM1_ESM.pdf]

Supplement 1

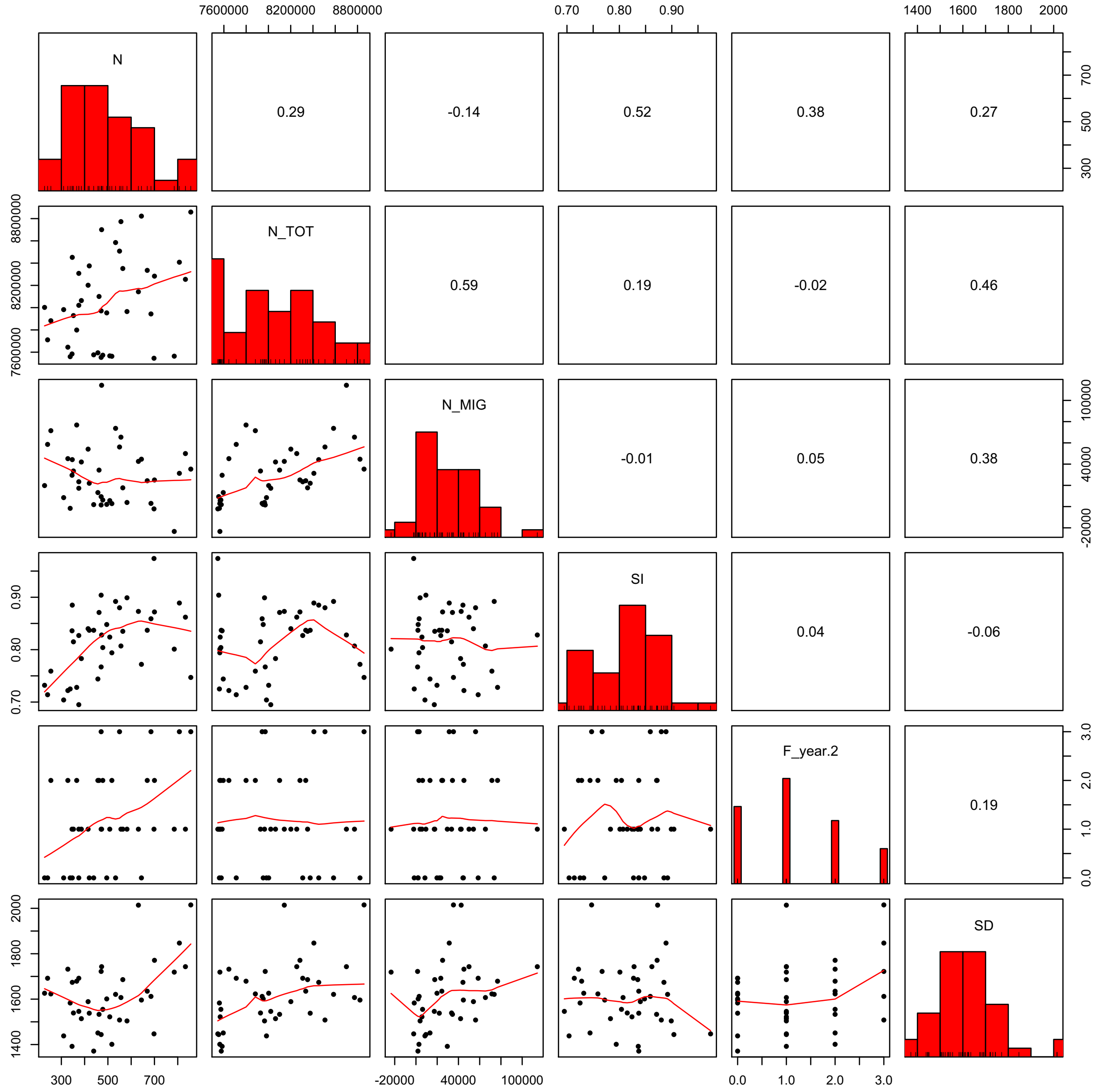

Figure S1: Frequency distributions (red bars) of the Austrian TBE incidence without vaccination N and the predictors used in the generalized linear models (GLMs): total human population N_{TOT}, net migration N_{MIG}, transformed Scandinavian index SI, beech fructification index 2 years prior F_{year-2} and, annual sunshine duration in hours SD. The following rank-order correlations with N have been determined: R=0.29 (N_{TOT}), R=-0.14 (N_{MIG}), R=0.52 (SI), R=0.38 (F_{year-2}), and R=0.27 (SD). Maximum collinearity of R=0.59 (N_{TOT} vs. N_{MIG}).

Supplement 2

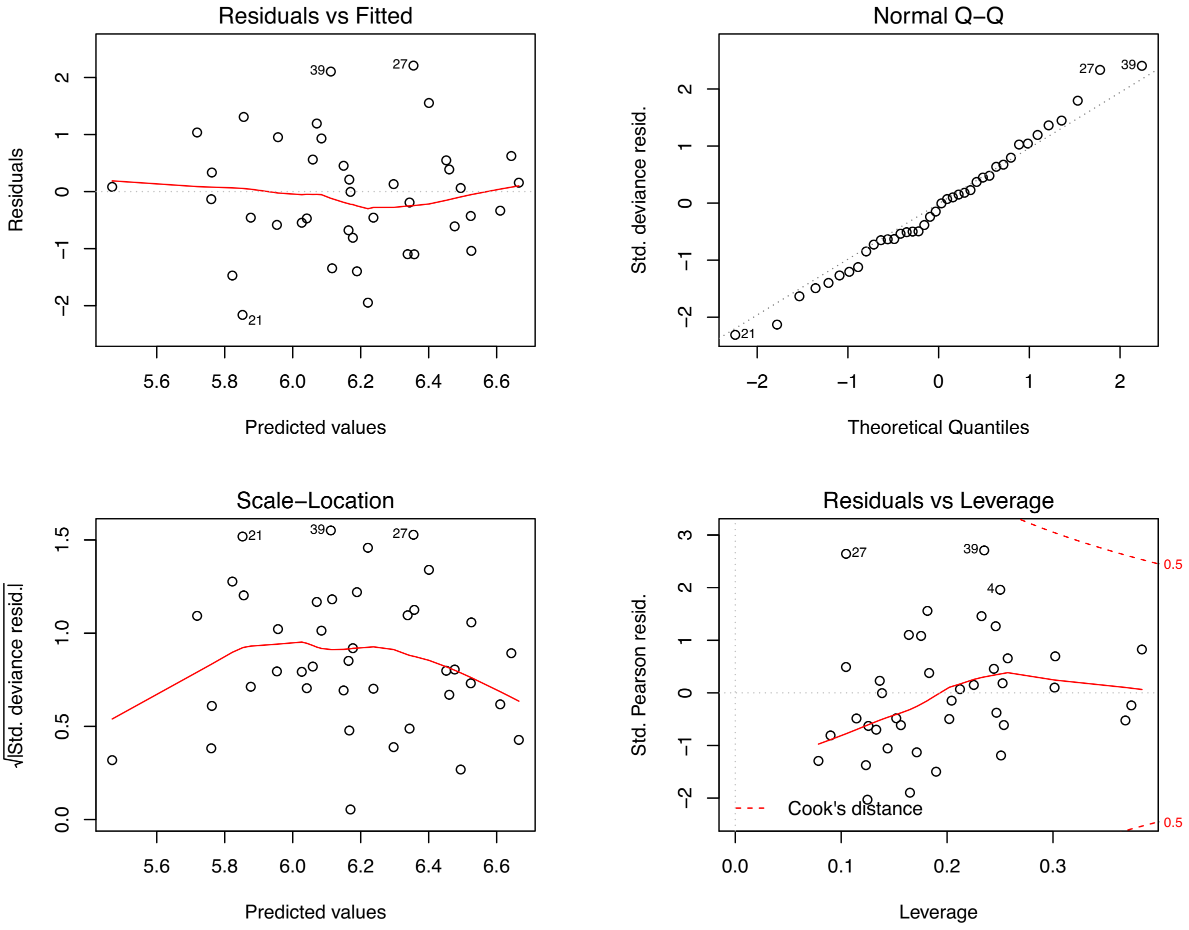

Figure S2: Statistical features of GLM4.

Supplement 3

Table S1: Akaike information criterion (AIC) and explained variance (R^2_{adj}) values for stepwise developed models GLM1–GLM4.

	AIC	R^2_{adj}
GLM1	519	0.10
GLM2	501	0.48
GLM3	490	0.66
GLM4	487	0.70
